# Supplementary material for: Linking Categorical and Dimensional Approaches to Assess Food-Related Emotions
Source: Foods. 2022 Mar 27;11(7):972. doi: 10.3390/foods11070972 (PMC8997768; doi:10.3390/foods11070972)
Supplement: Supplementary file 1 [file foods-11-00972-s001.zip › supplementary material/Supplementary file S2 Word_Mapping_Distributions.pdf]

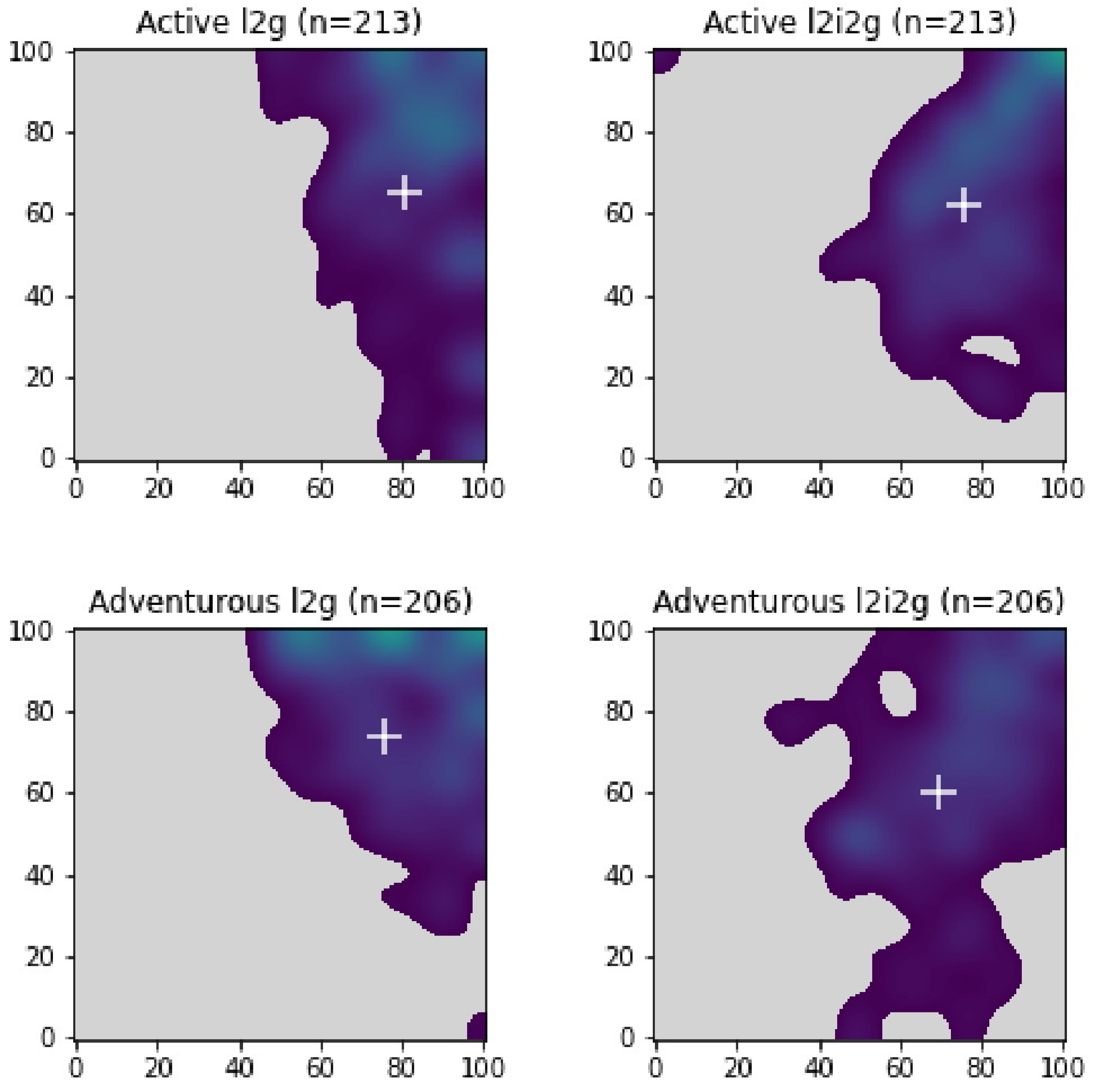
Active l2g (n=213)
Active l2i2g (n=213)
Adventurous l2g (n=206)
Adventurous l2i2g (n=206)
0
20
40
60
80
100

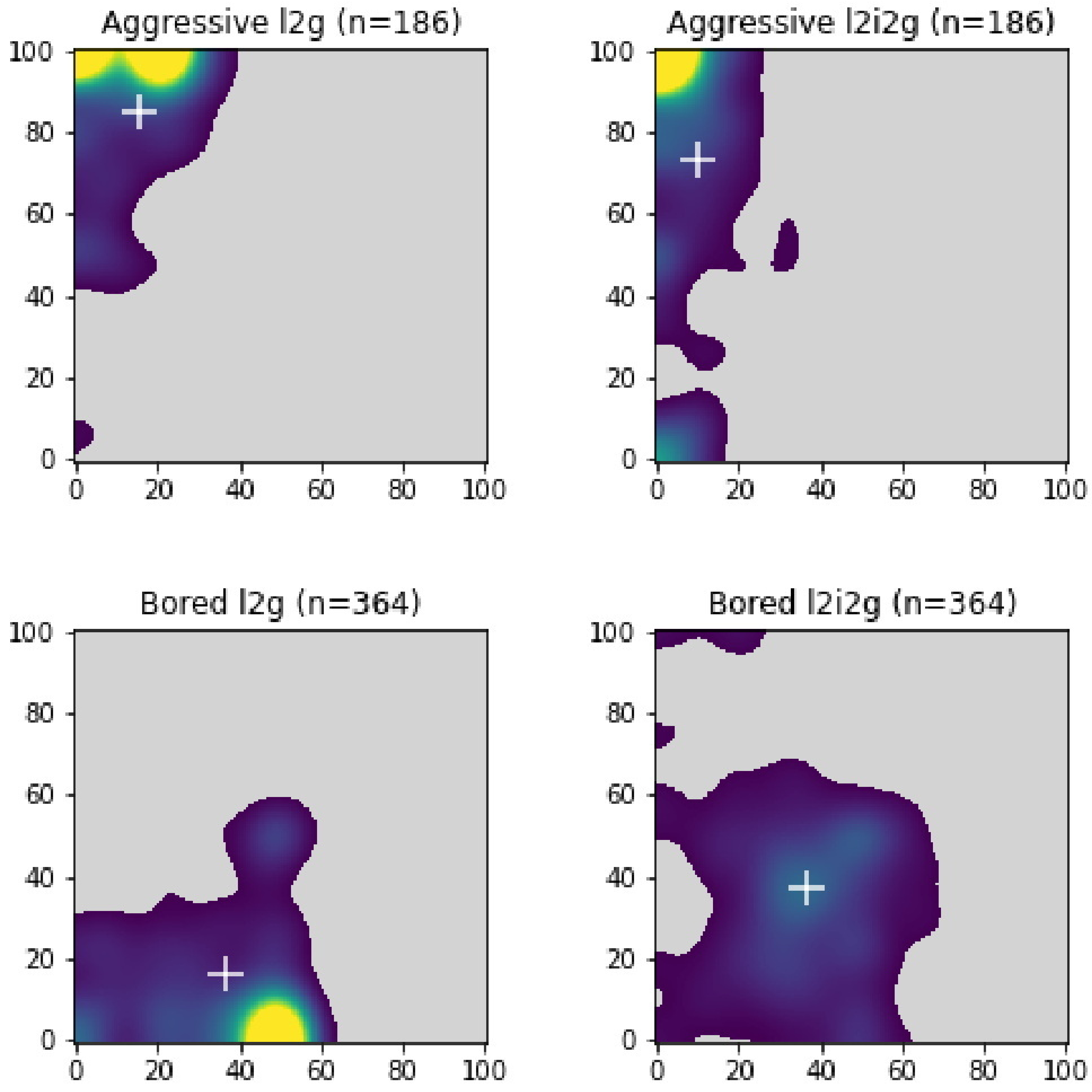
Aggressive l2g (n=186)
Aggressive l2i2g (n=186)
Bored l2g (n=364)
Bored l2i2g (n=364)
0
20
40
60
80
100

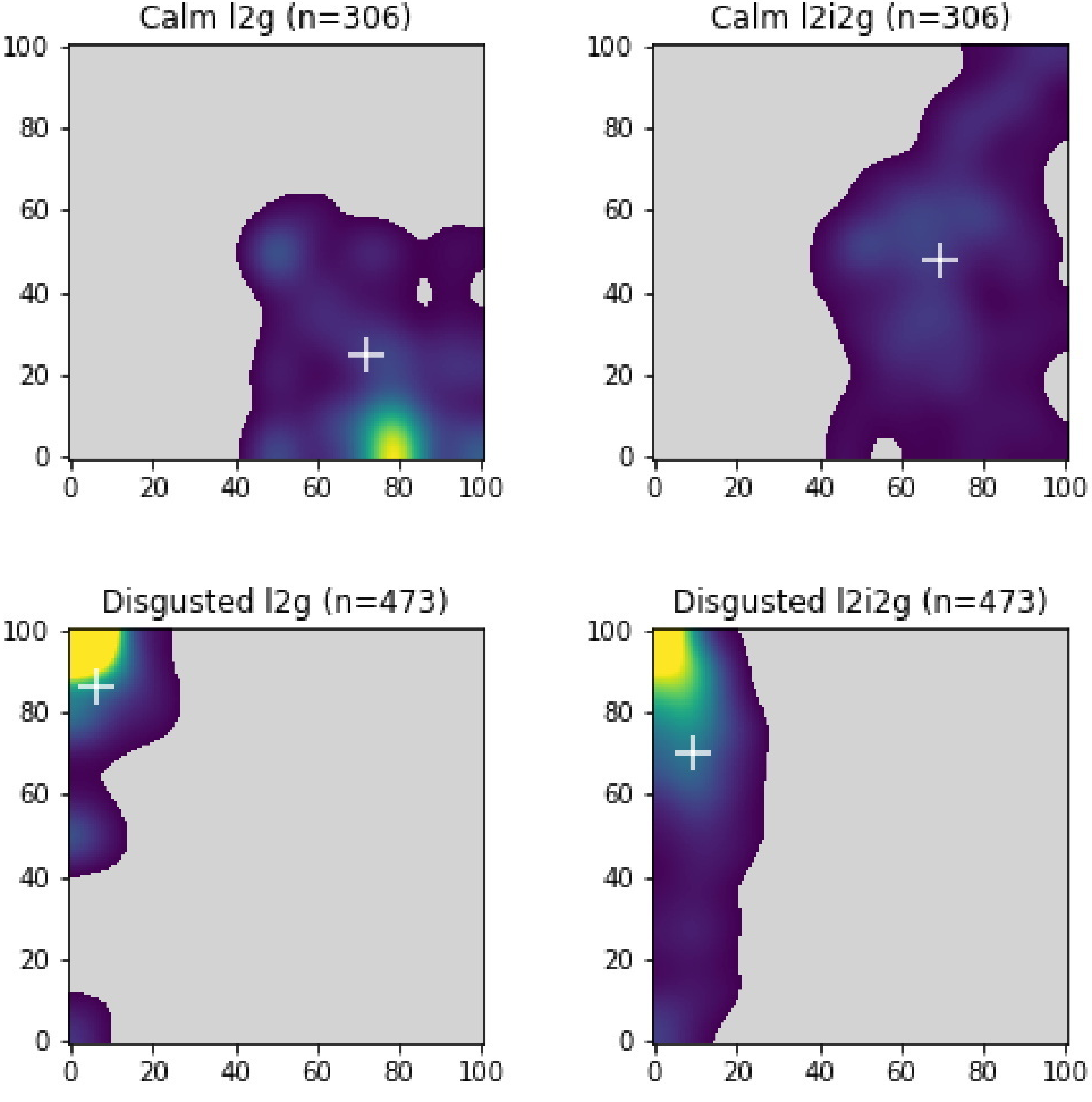
Calm l2g (n=306)
Calm l2i2g (n=306)
Disgusted l2g (n=473)
Disgusted l2i2g (n=473)
0
20
40
60
80
100

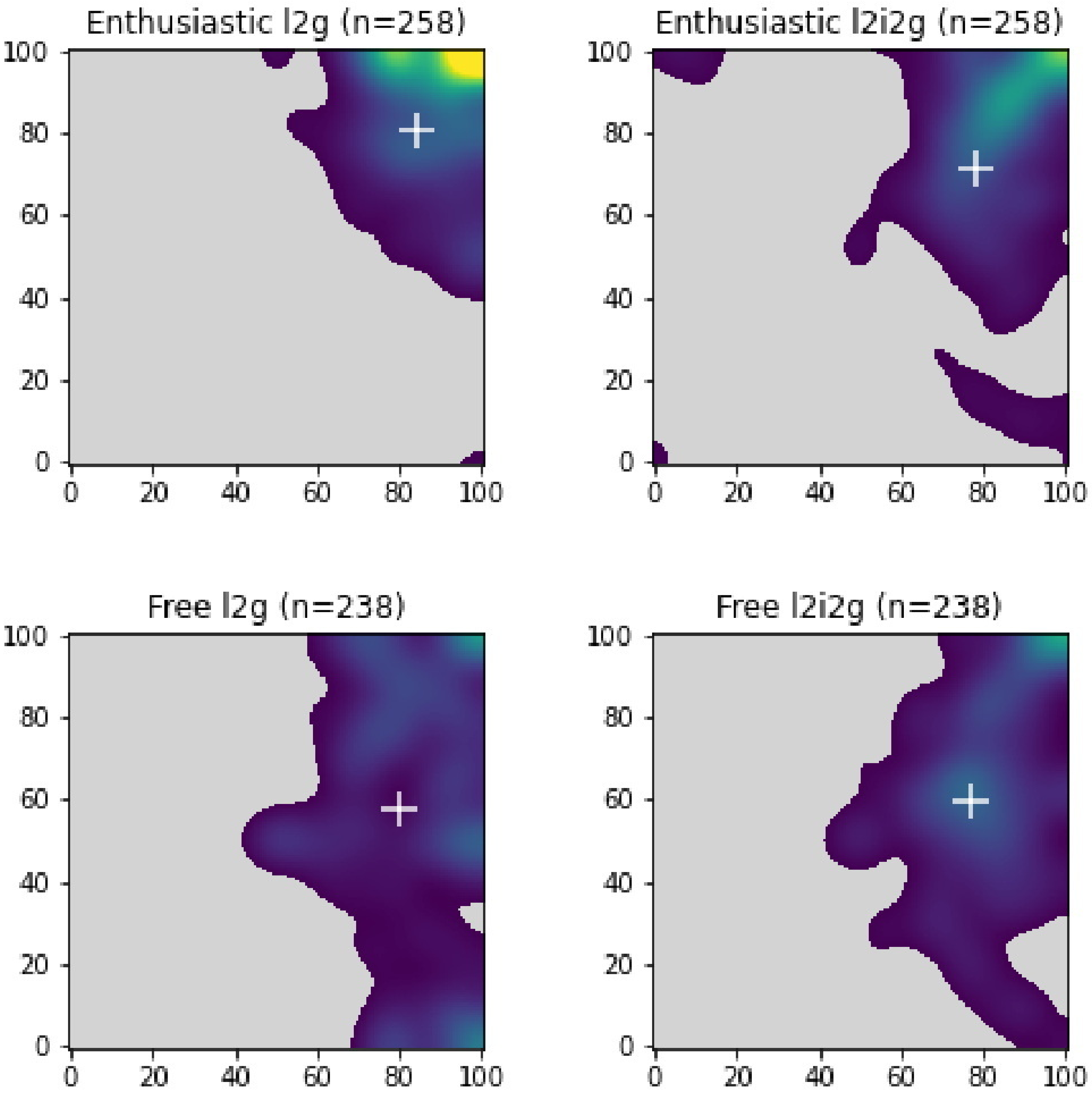
Enthusiastic l2g (n=258)
Enthusiastic l2i2g (n=258)
Free l2g (n=238)
Free l2i2g (n=238)
0
20
40
60
80
100

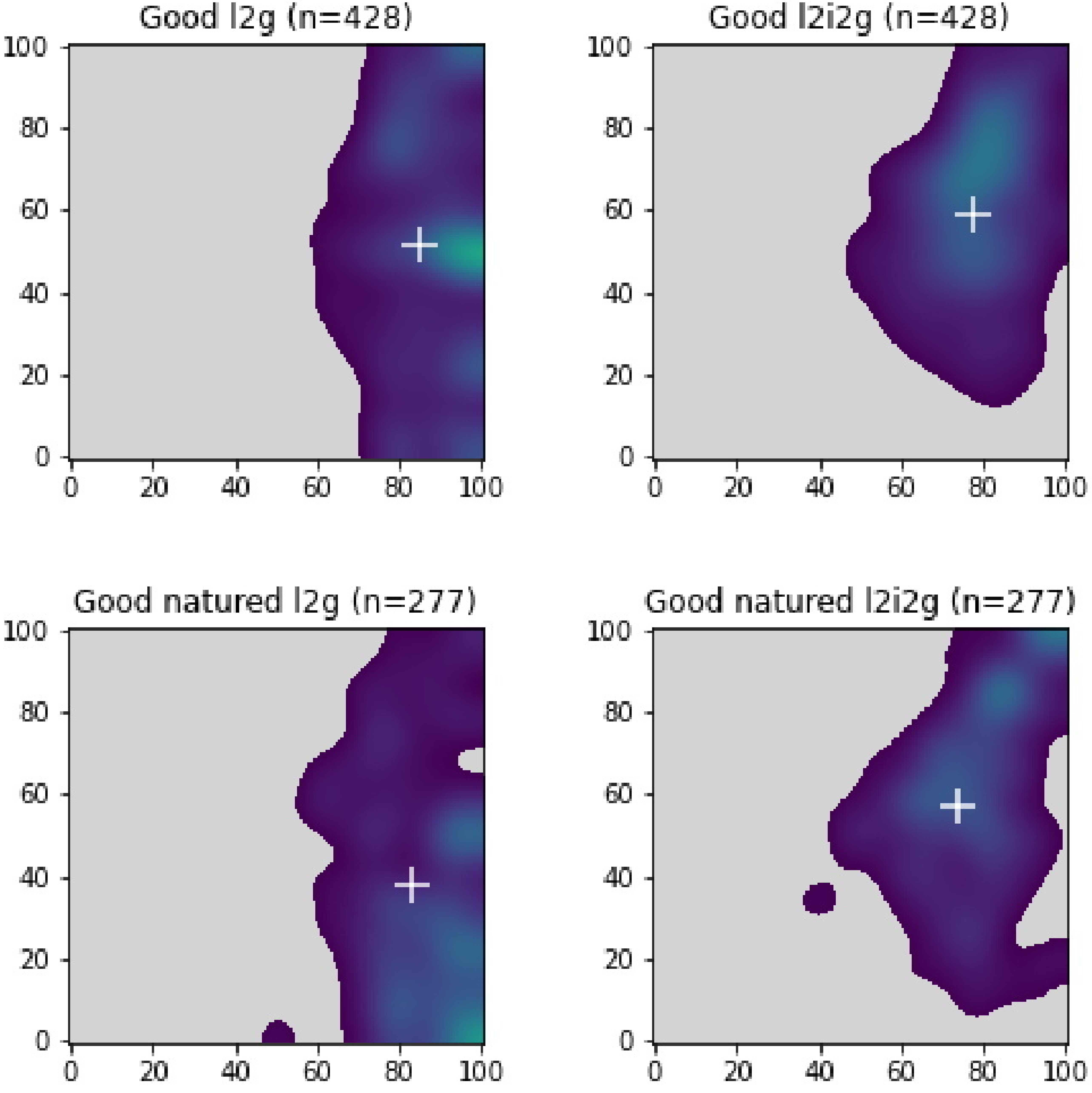
Good l2g (n=428)
Good l2i2g (n=428)
Good natured l2g (n=277)
Good natured l2i2g (n=277)
0
20
40
60
80
100

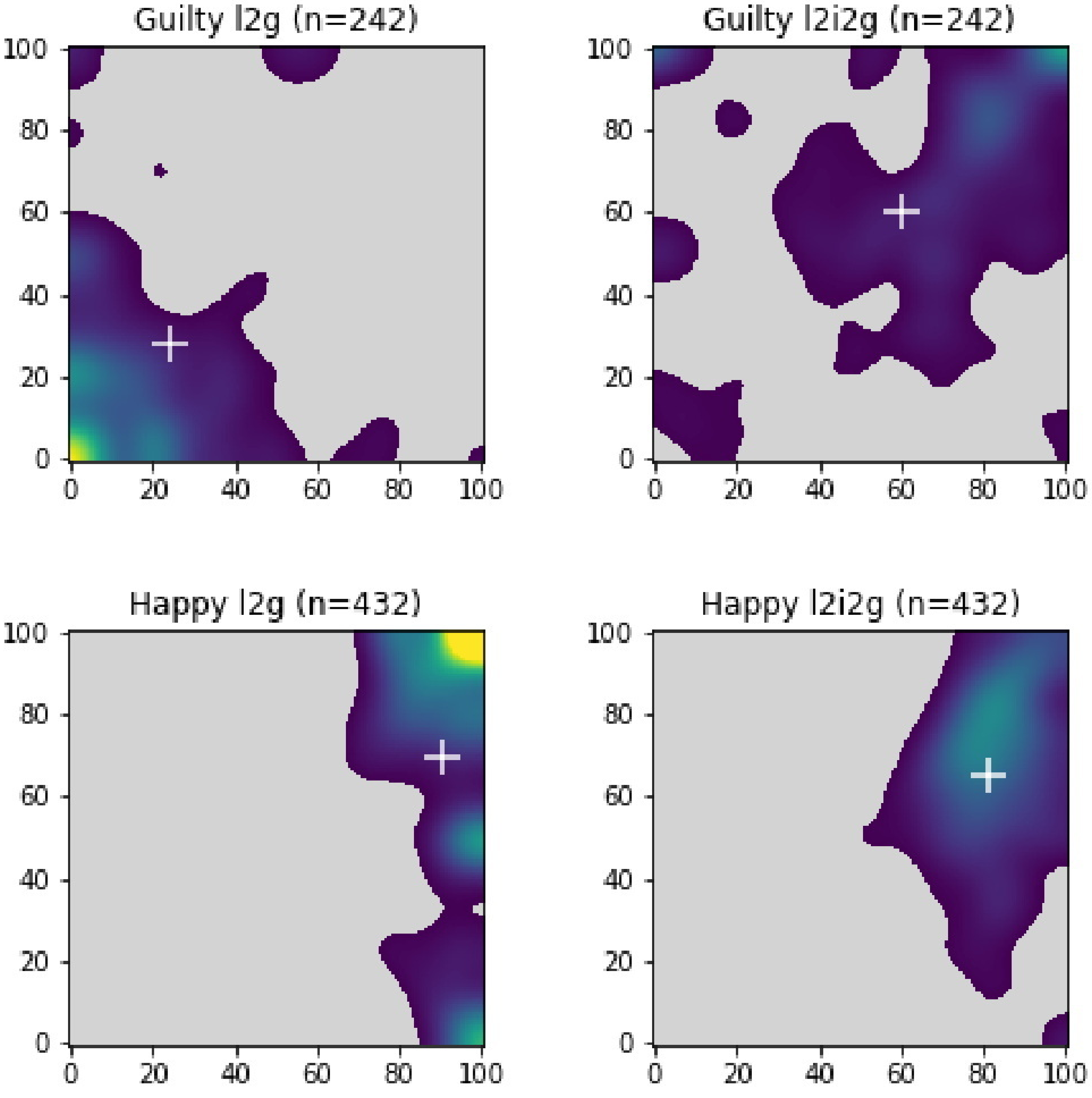
Guilty l2g (n=242)
Guilty l2i2g (n=242)
Happy l2g (n=432)
Happy l2i2g (n=432)
0
20
40
60
80
100

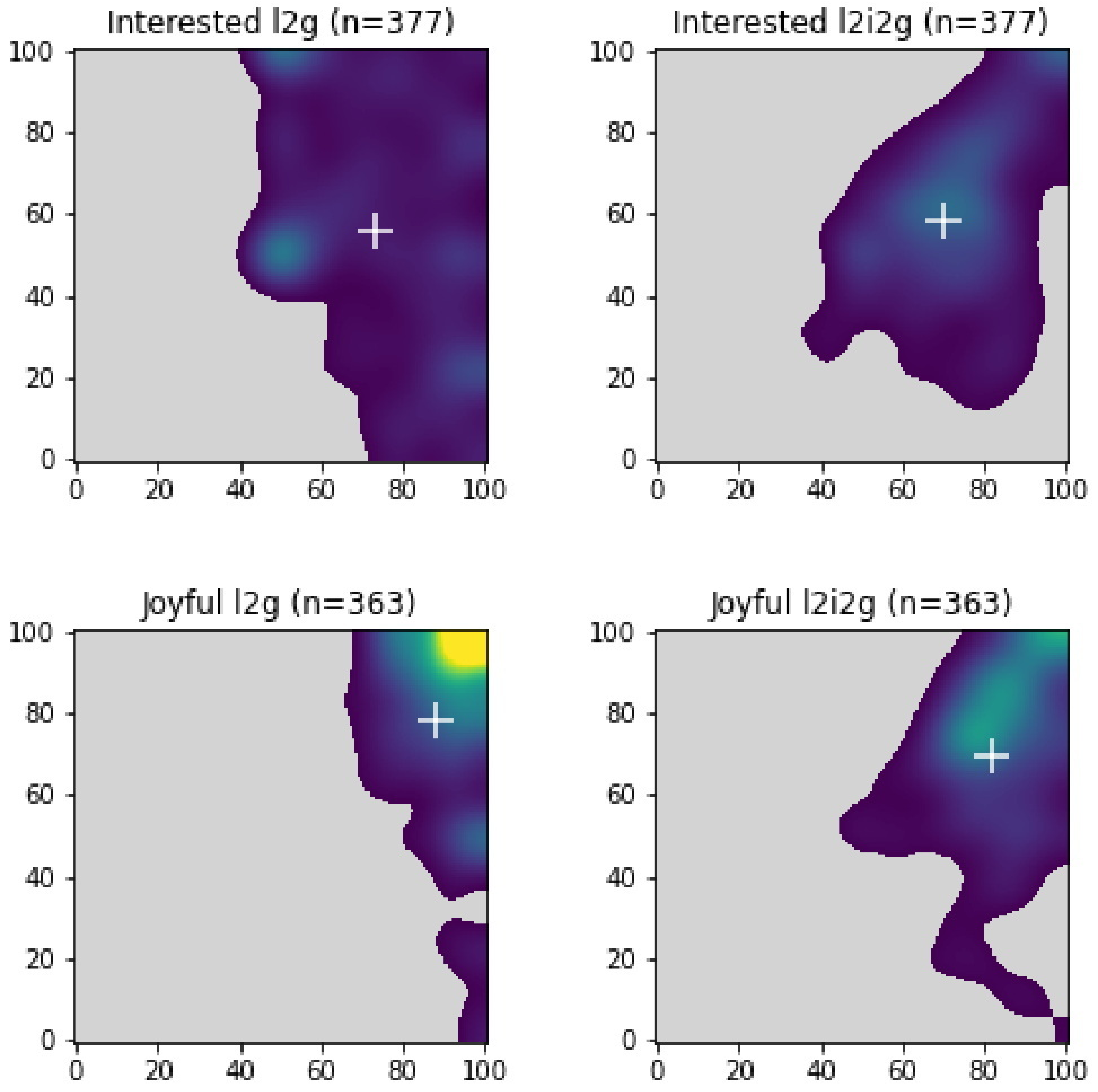

Interested I2g (n=377)
Interested I2i2g (n=377)
Joyful I2g (n=363)
Joyful I2i2g (n=363)
0
20
40
60
80
100

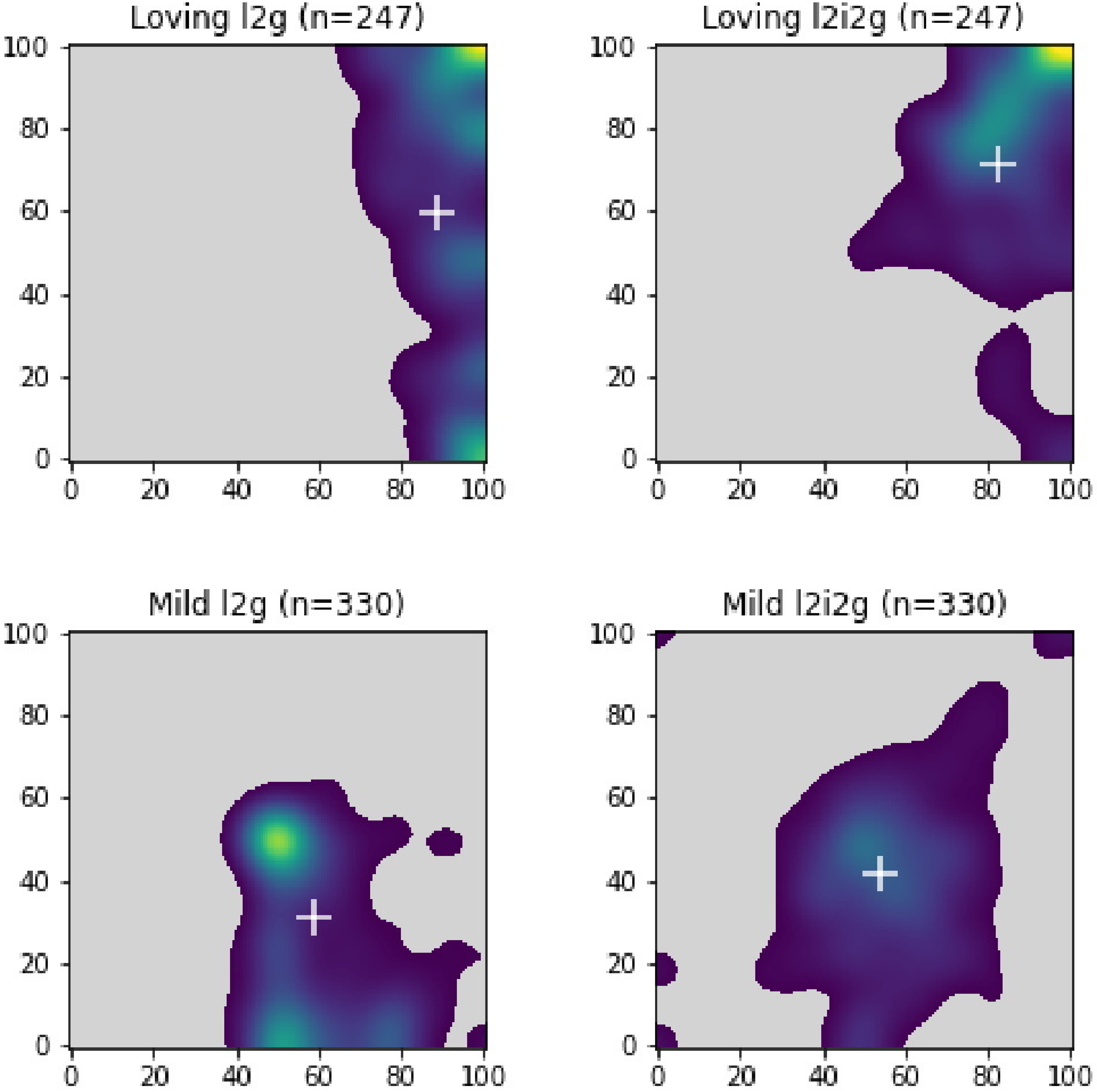

Loving l2g (n=247)
Loving l2i2g (n=247)
Mild l2g (n=330)
Mild l2i2g (n=330)
0
20
40
60
80
100

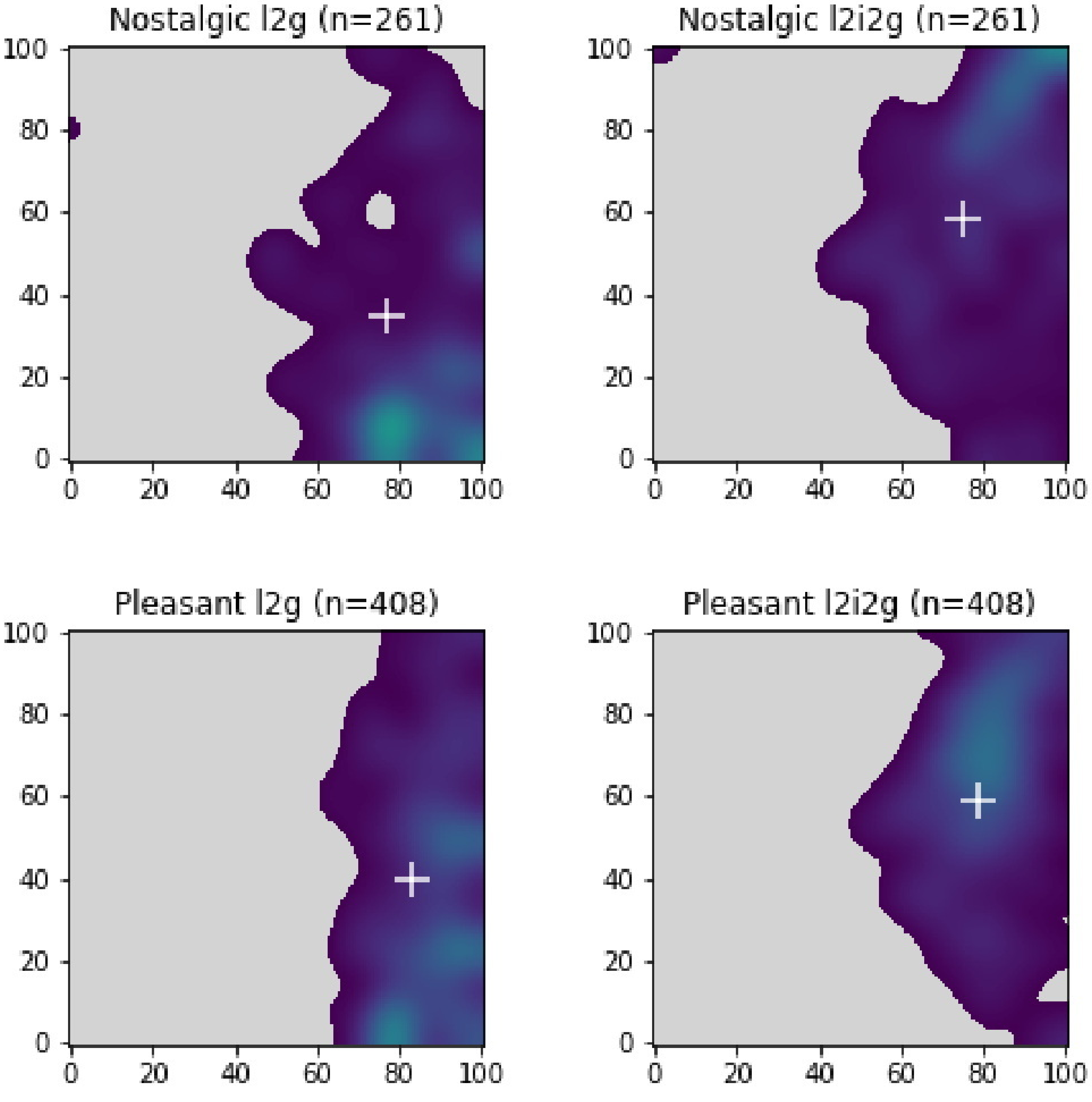
Nostalgic l2g (n=261)
Nostalgic l2i2g (n=261)
Pleasant l2g (n=408)
Pleasant l2i2g (n=408)
0
20
40
60
80
100

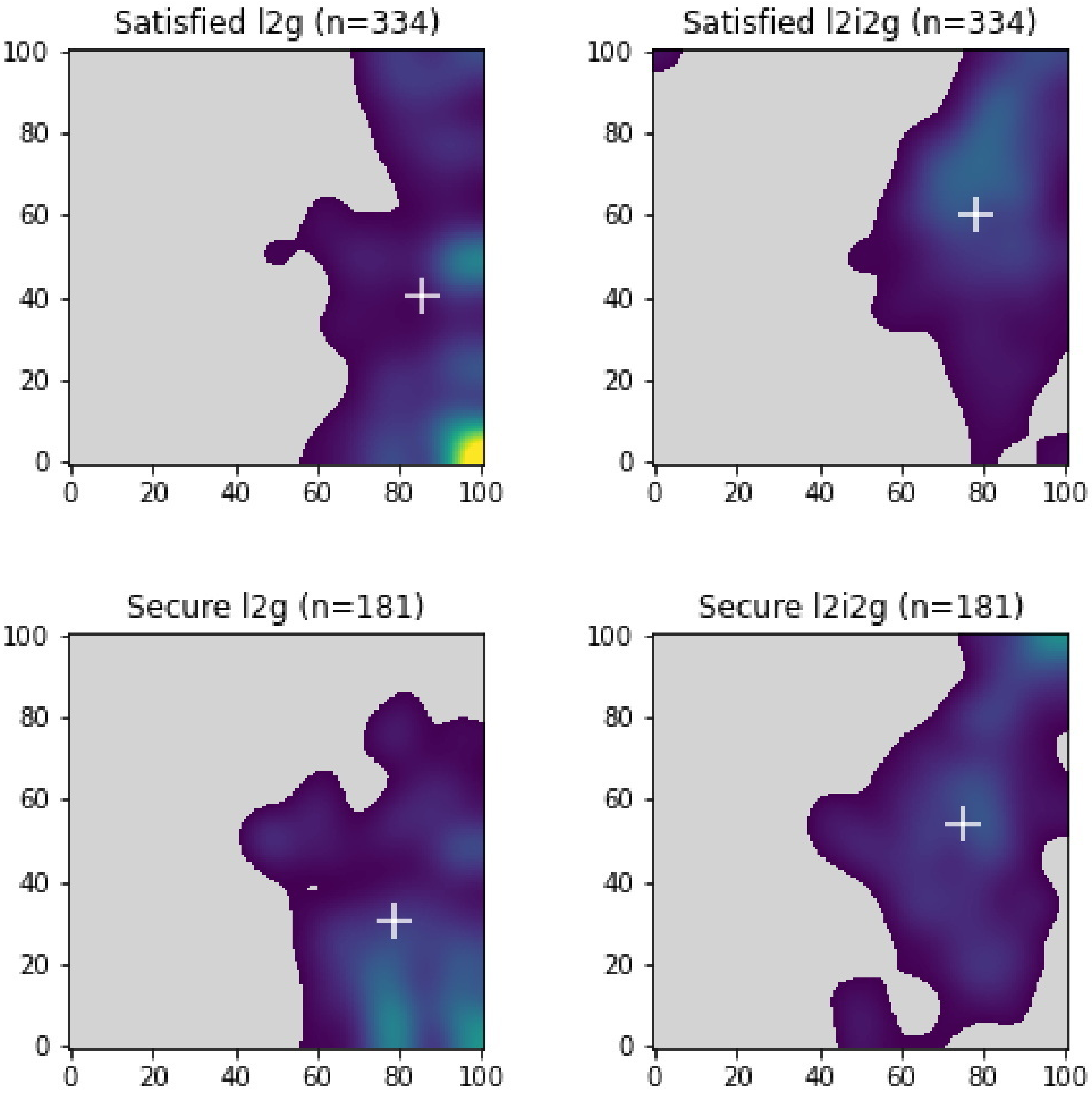
Satisfied l2g (n=334)
Satisfied l2i2g (n=334)
Secure l2g (n=181)
Secure l2i2g (n=181)
0
20
40
60
80
100

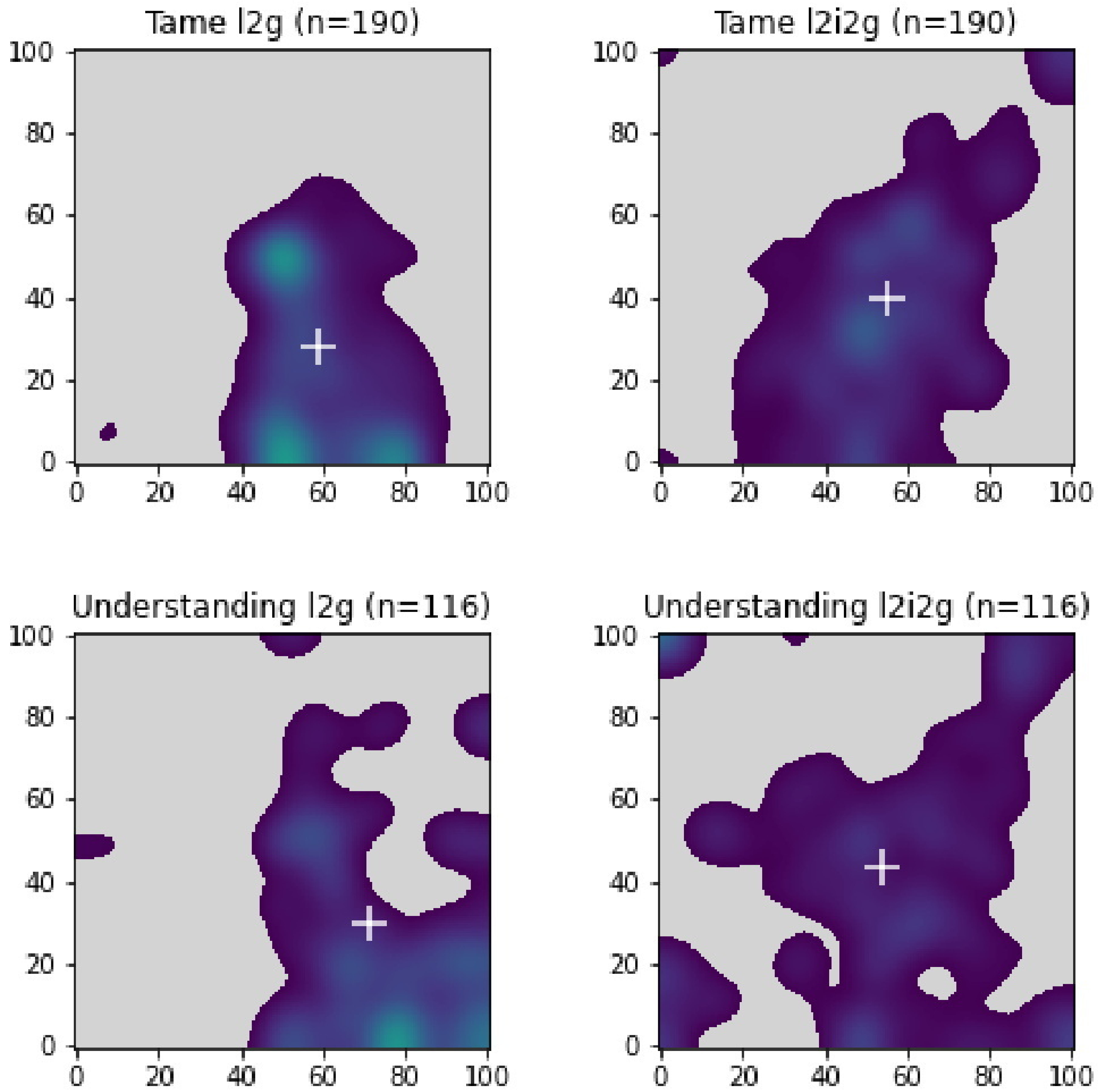
Tame l2g (n=190)
Tame l2i2g (n=190)
Understanding l2g (n=116)
Understanding l2i2g (n=116)
0
20
40
60
80
100

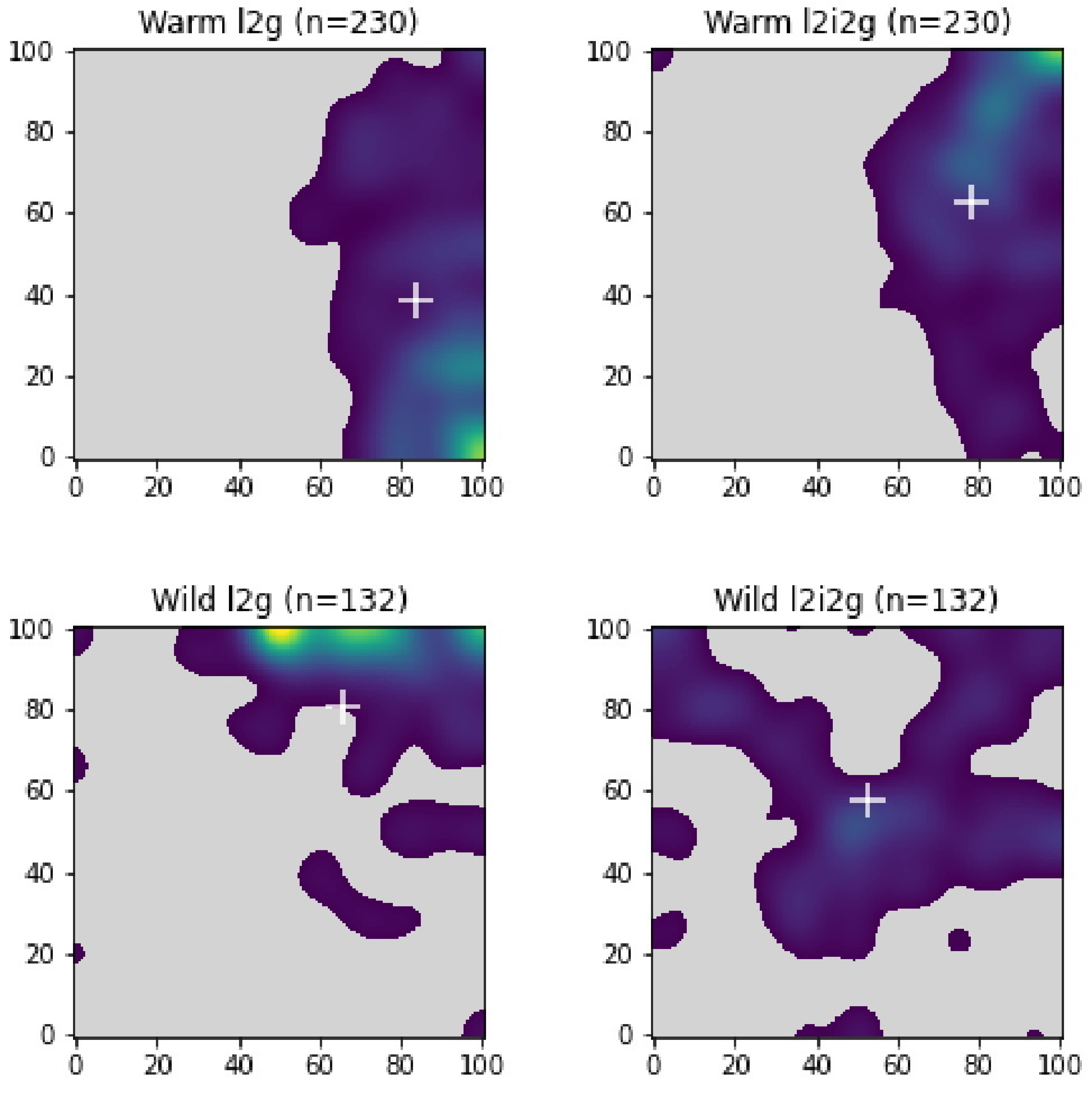
Warm l2g (n=230)
Warm l2i2g (n=230)
Wild l2g (n=132)
Wild l2i2g (n=132)
0
20
40
60
80
100

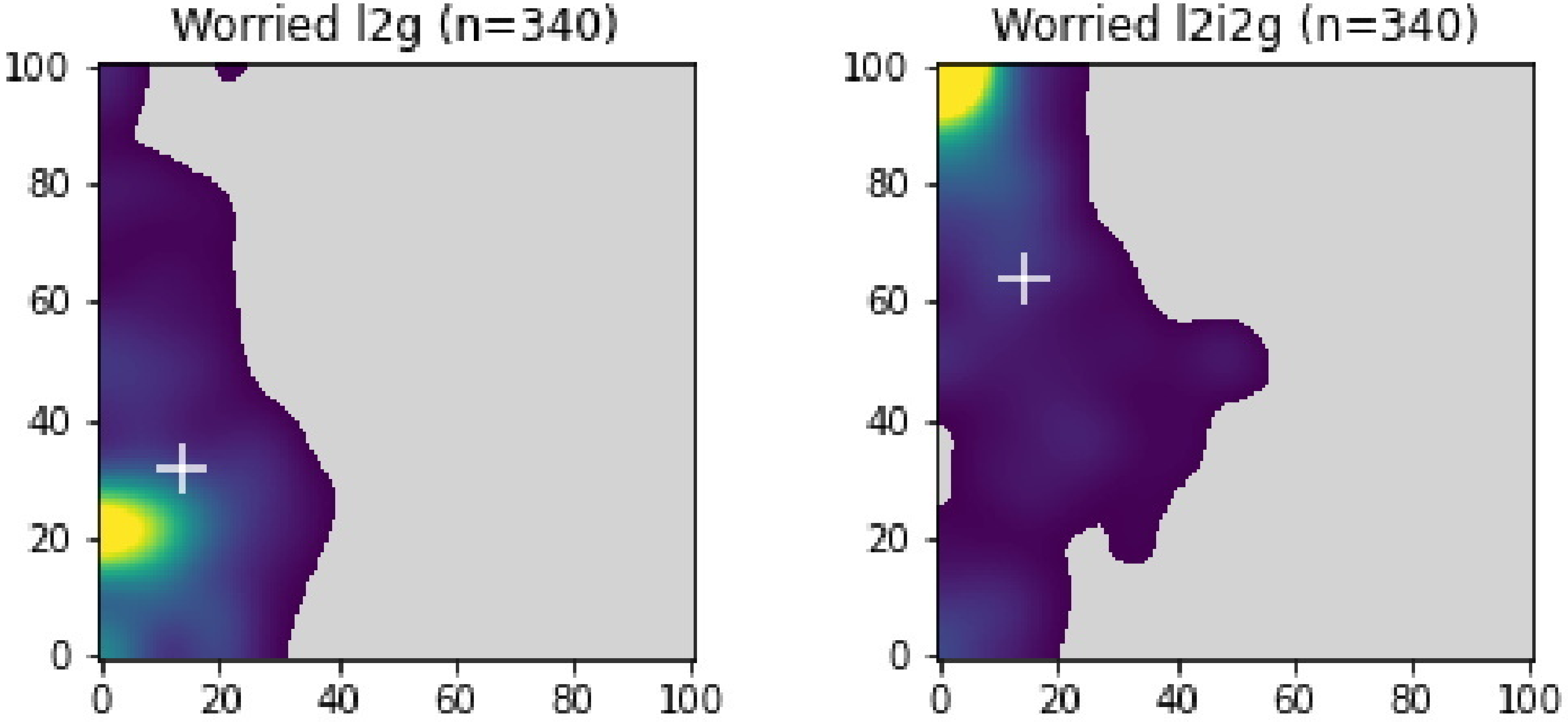

Worried l2g (n=340)
Worried l2i2g (n=340)
0
20
40
60
80
100
