# Supplementary figures and images for: Linking Categorical and Dimensional Approaches to Assess Food-Related Emotions
Source: Foods. 2022 Mar 27;11(7):972. doi: 10.3390/foods11070972 (PMC8997768; doi:10.3390/foods11070972)

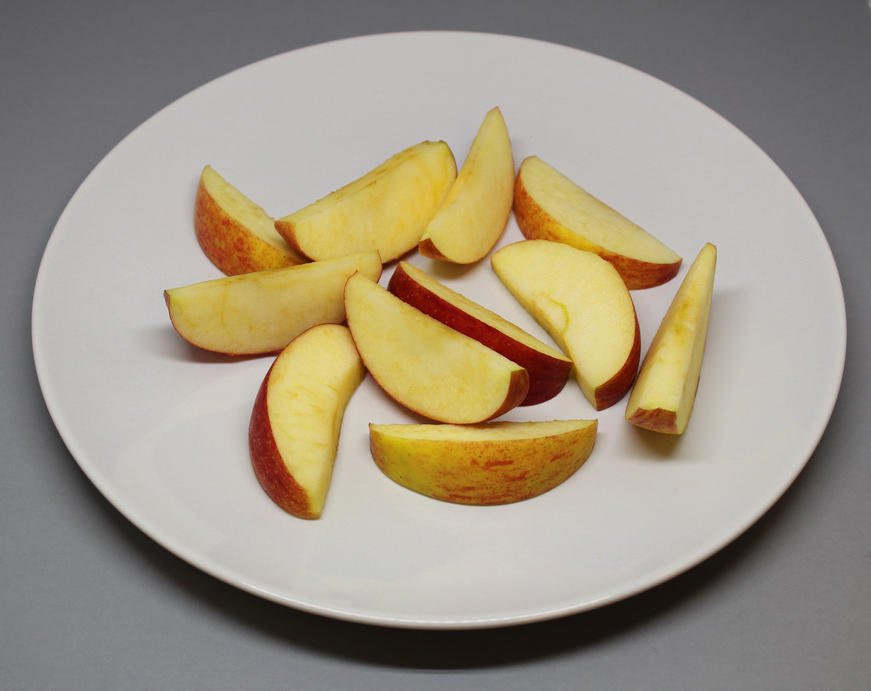

Supplement: Supplementary file 1 [file foods-11-00972-s001.zip › supplementary material/supplementary file S1/1.jpg]

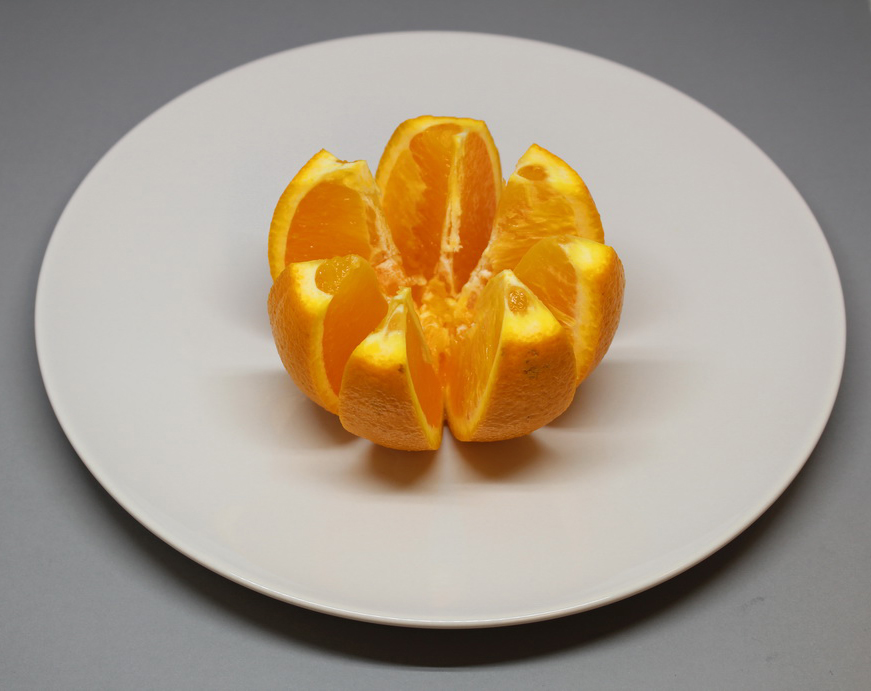

Supplement: Supplementary file 1 [file foods-11-00972-s001.zip › supplementary material/supplementary file S1/10.jpg]

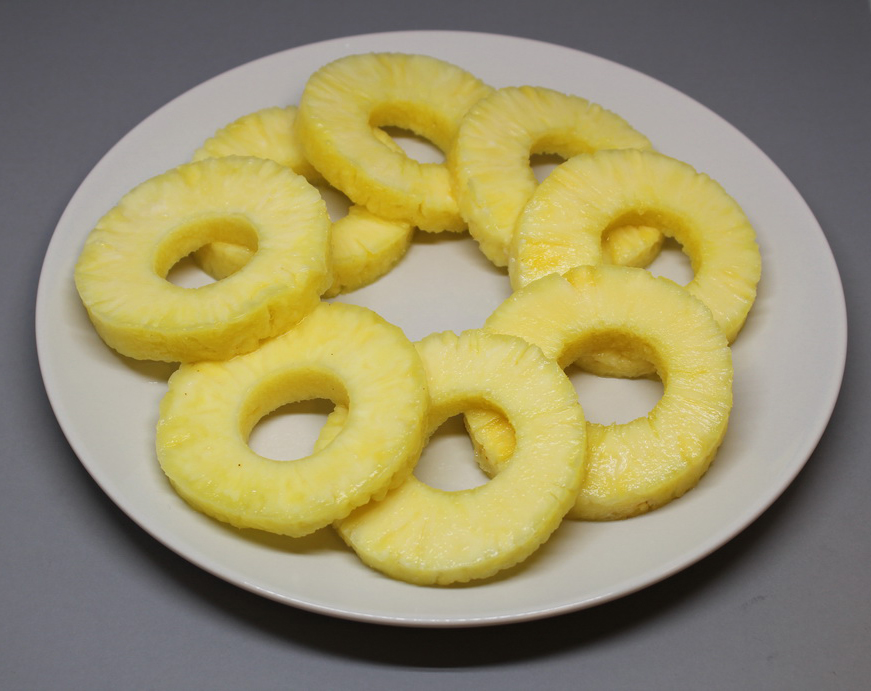

Supplement: Supplementary file 1 [file foods-11-00972-s001.zip › supplementary material/supplementary file S1/11.jpg]

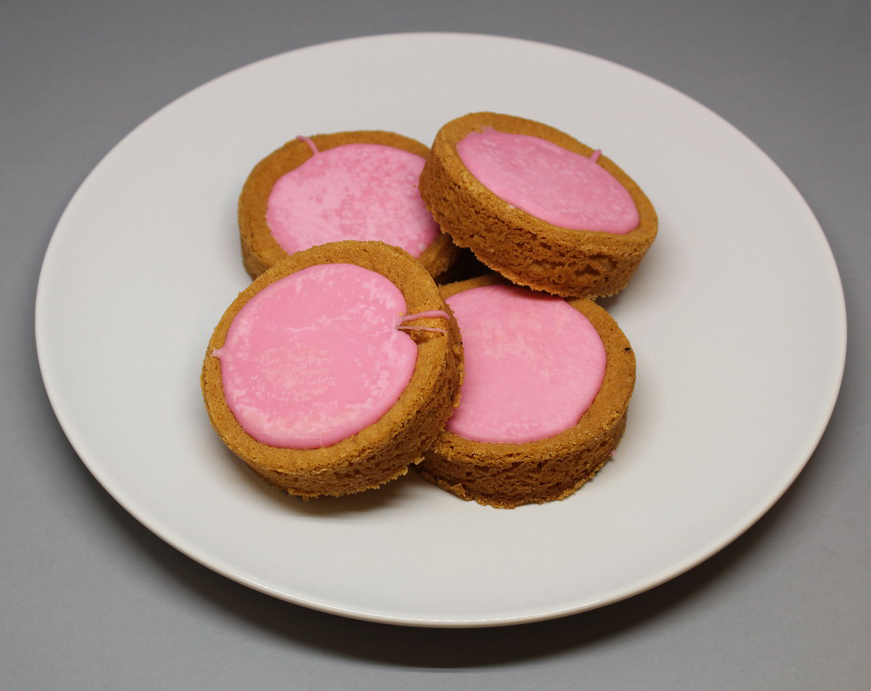

Supplement: Supplementary file 1 [file foods-11-00972-s001.zip › supplementary material/supplementary file S1/12.jpg]

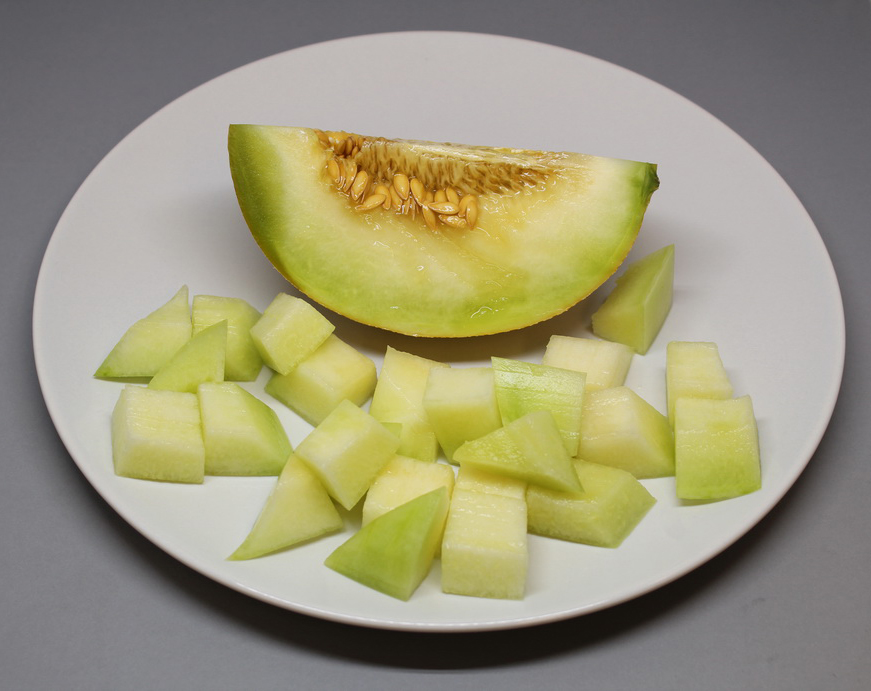

Supplement: Supplementary file 1 [file foods-11-00972-s001.zip › supplementary material/supplementary file S1/15.jpg]

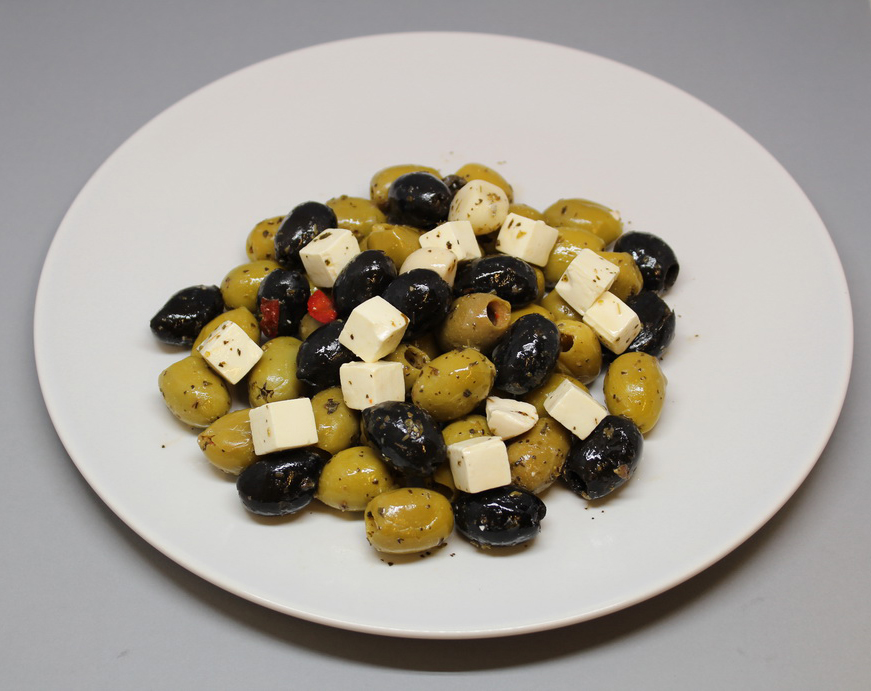

Supplement: Supplementary file 1 [file foods-11-00972-s001.zip › supplementary material/supplementary file S1/18.jpg]

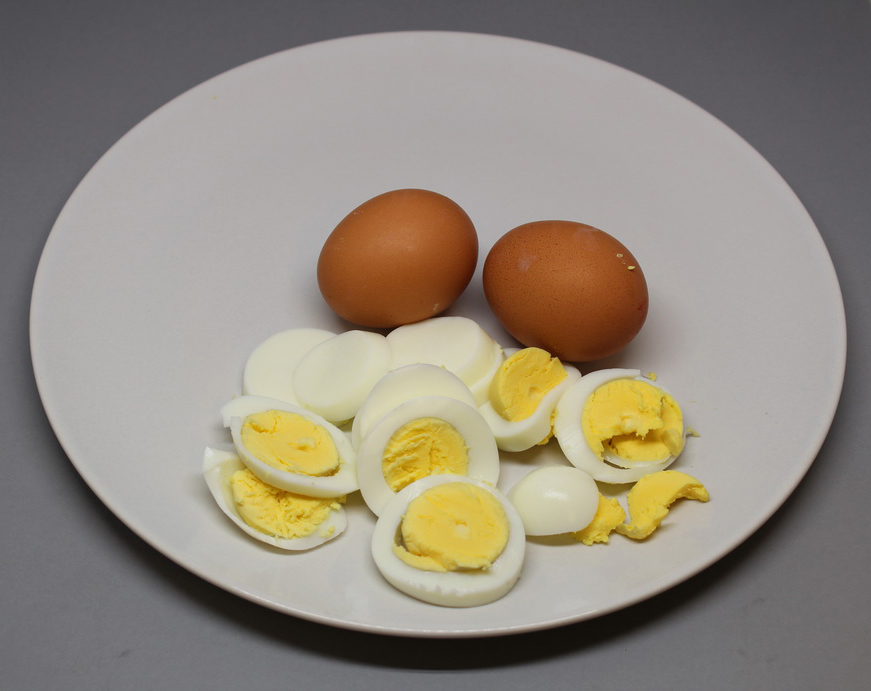

Supplement: Supplementary file 1 [file foods-11-00972-s001.zip › supplementary material/supplementary file S1/20.jpg]

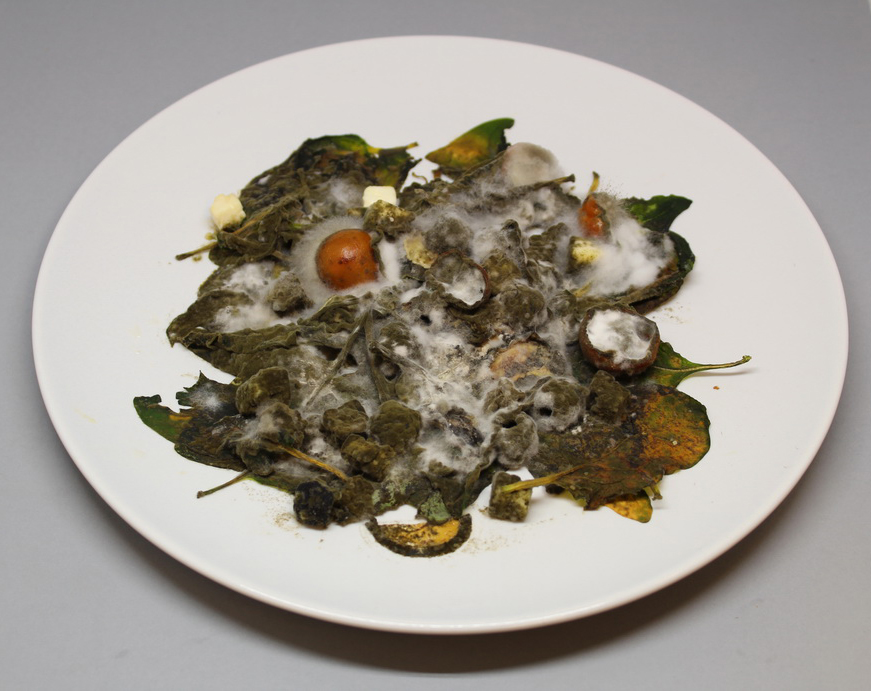

Supplement: Supplementary file 1 [file foods-11-00972-s001.zip › supplementary material/supplementary file S1/32.jpg]

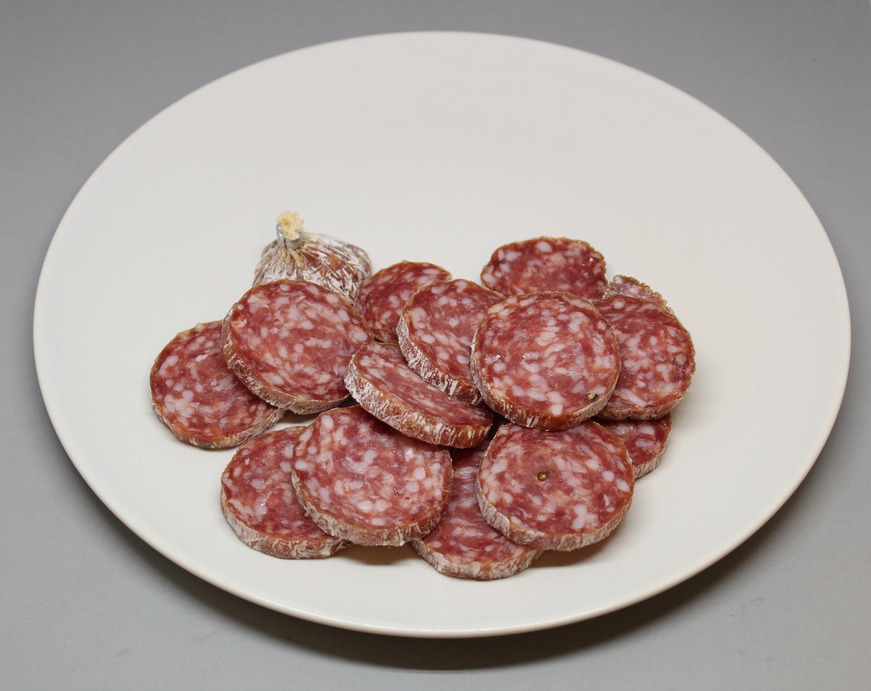

Supplement: Supplementary file 1 [file foods-11-00972-s001.zip › supplementary material/supplementary file S1/34.jpg]

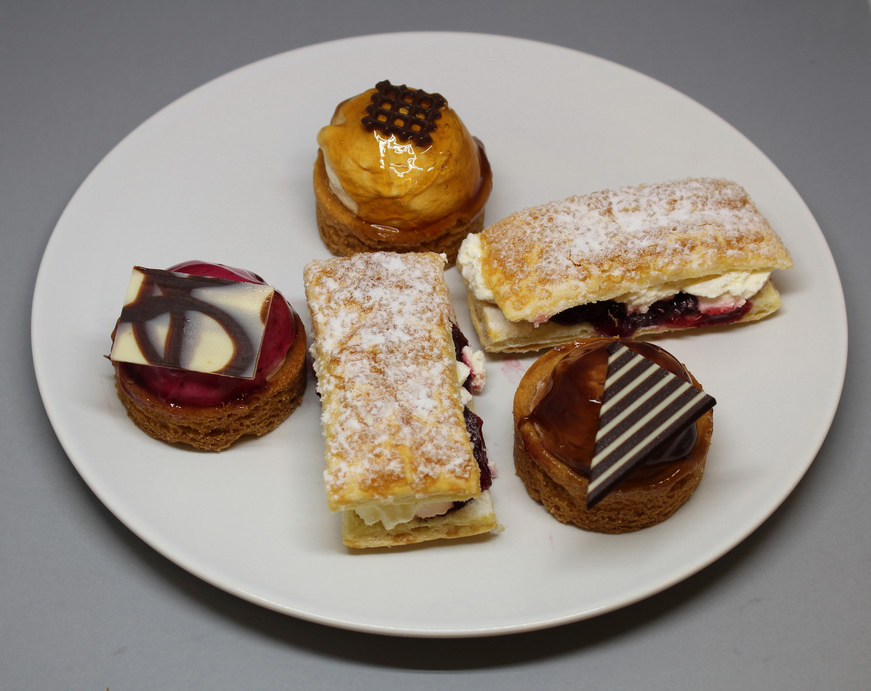

Supplement: Supplementary file 1 [file foods-11-00972-s001.zip › supplementary material/supplementary file S1/35.jpg]

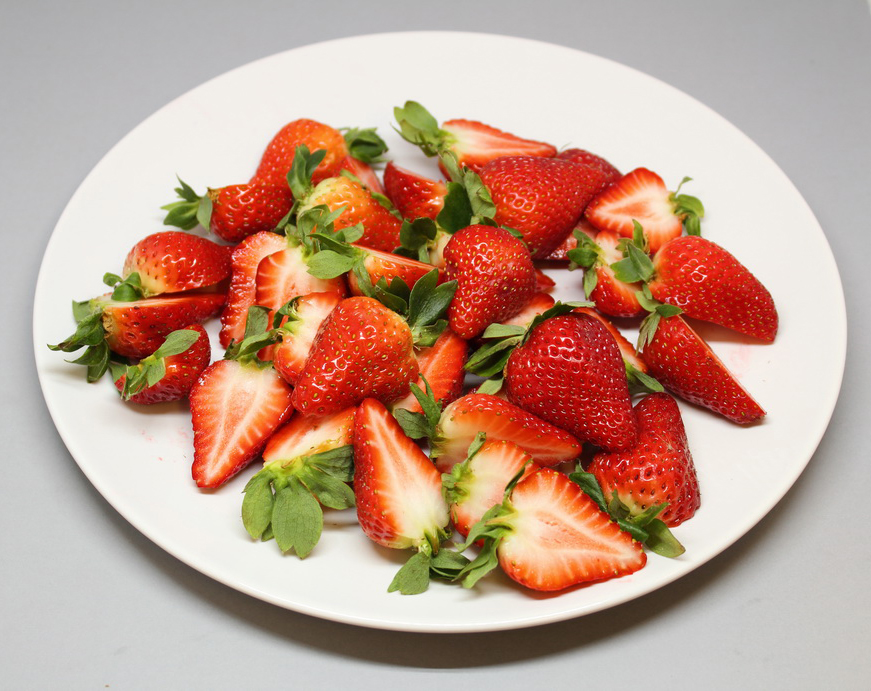

Supplement: Supplementary file 1 [file foods-11-00972-s001.zip › supplementary material/supplementary file S1/36.jpg]

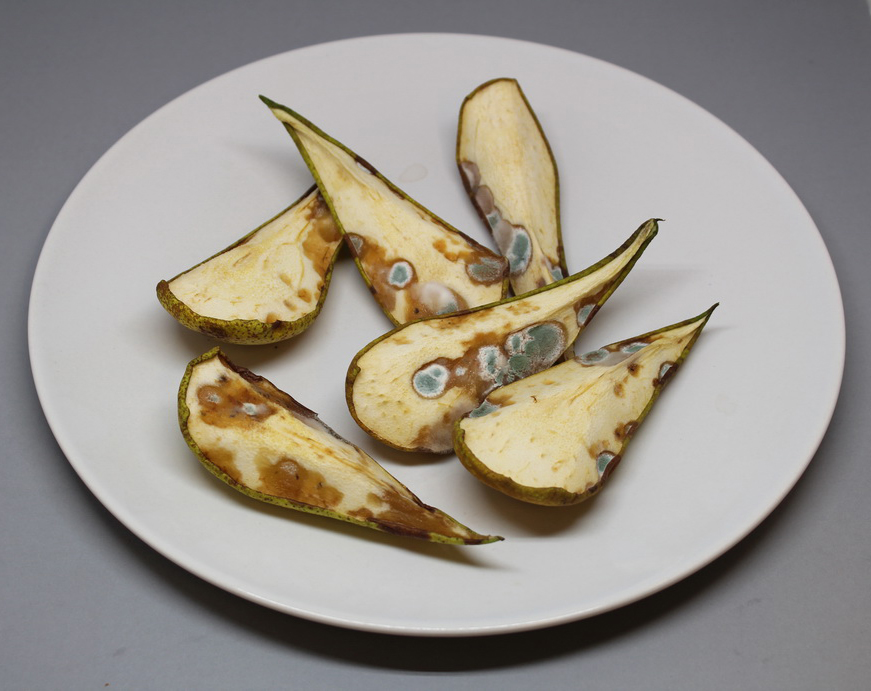

Supplement: Supplementary file 1 [file foods-11-00972-s001.zip › supplementary material/supplementary file S1/37.jpg]

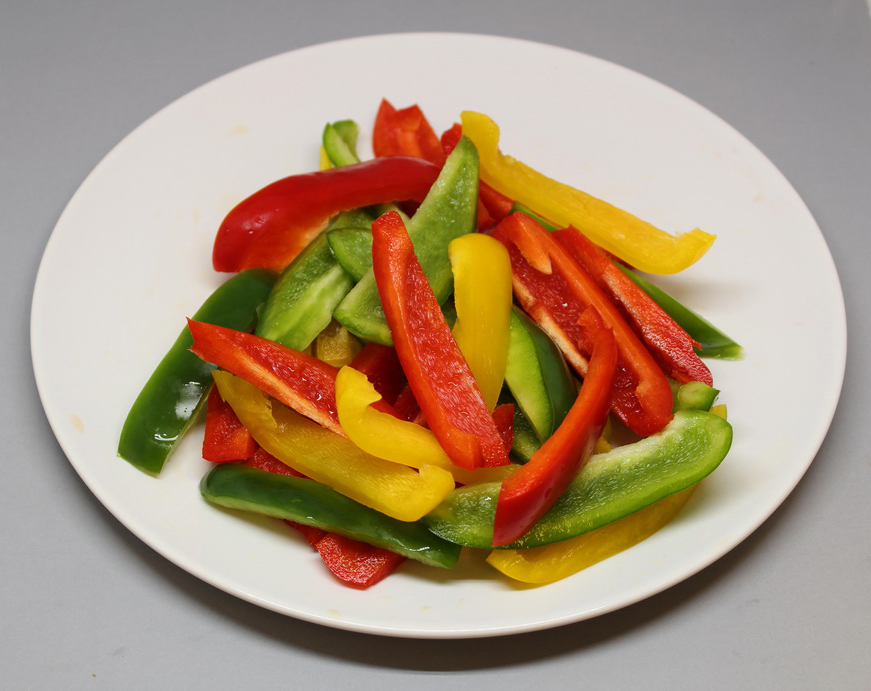

Supplement: Supplementary file 1 [file foods-11-00972-s001.zip › supplementary material/supplementary file S1/39.jpg]

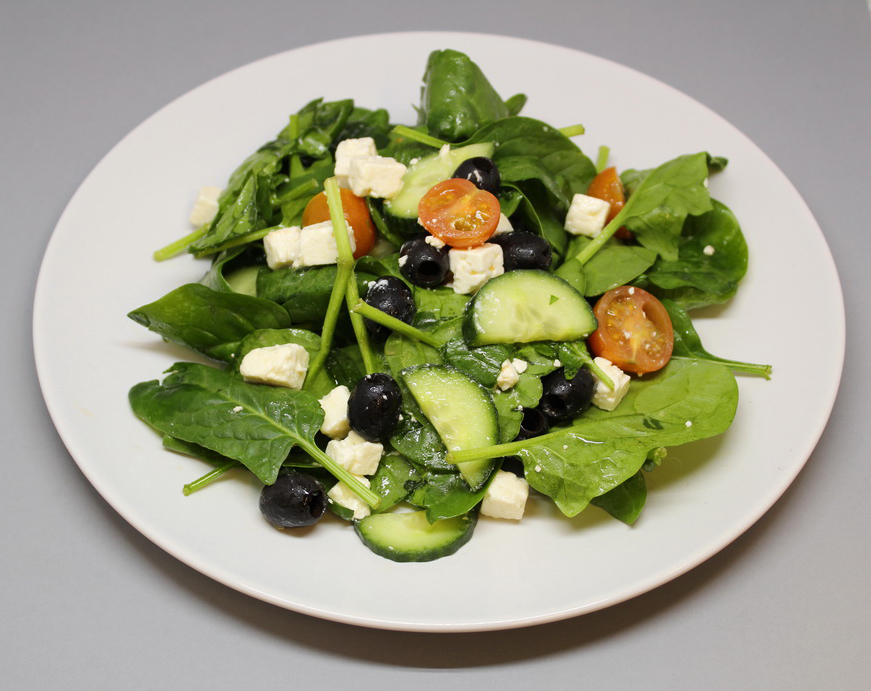

Supplement: Supplementary file 1 [file foods-11-00972-s001.zip › supplementary material/supplementary file S1/4.jpg]

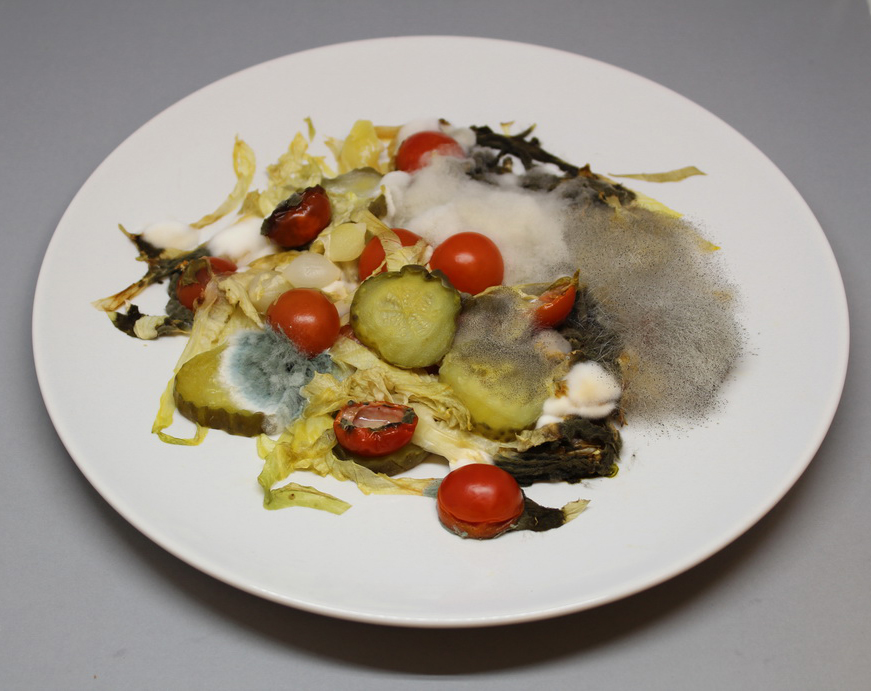

Supplement: Supplementary file 1 [file foods-11-00972-s001.zip › supplementary material/supplementary file S1/41.jpg]

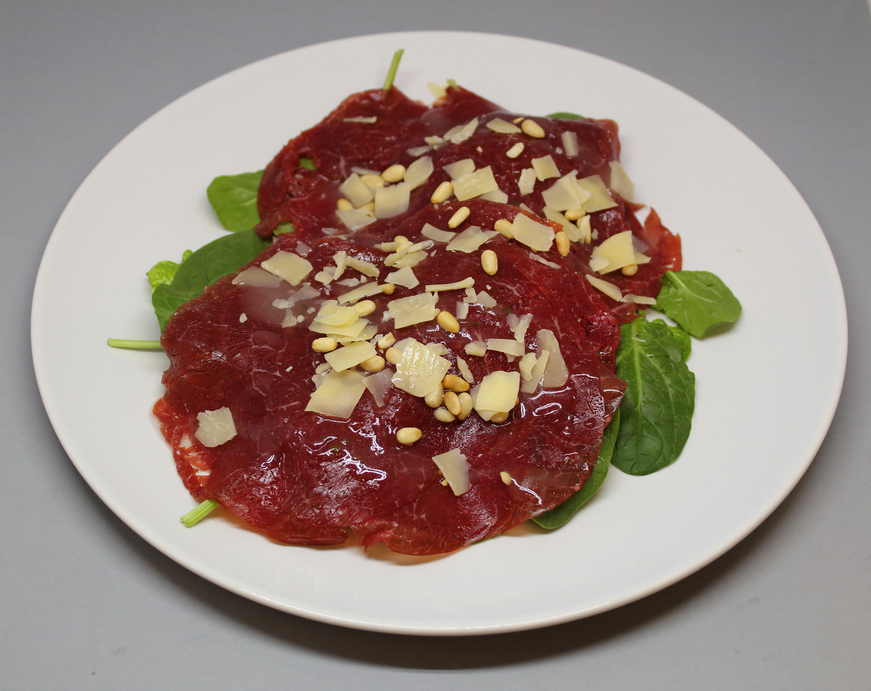

Supplement: Supplementary file 1 [file foods-11-00972-s001.zip › supplementary material/supplementary file S1/44.jpg]

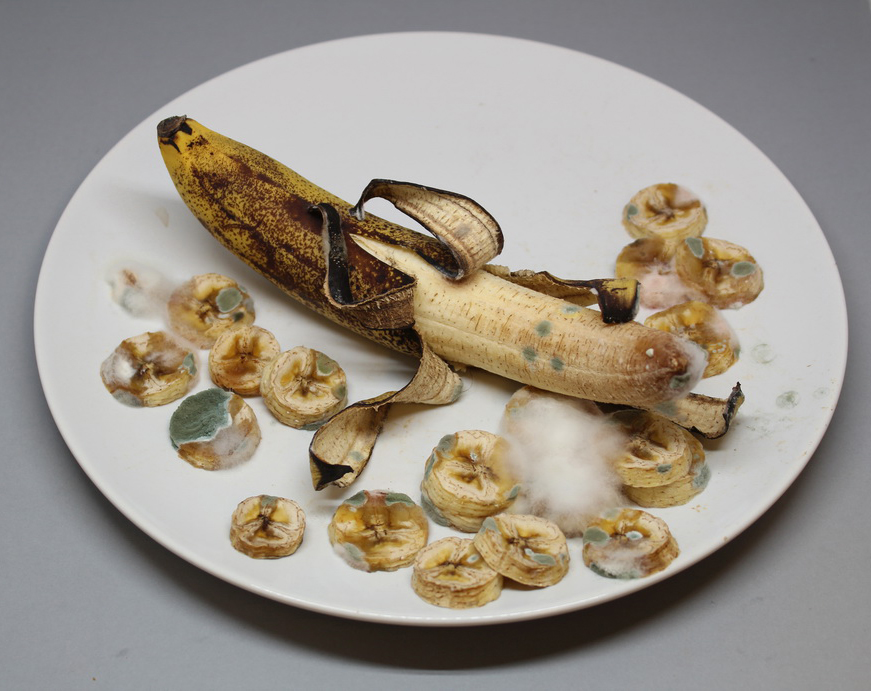

Supplement: Supplementary file 1 [file foods-11-00972-s001.zip › supplementary material/supplementary file S1/47.jpg]

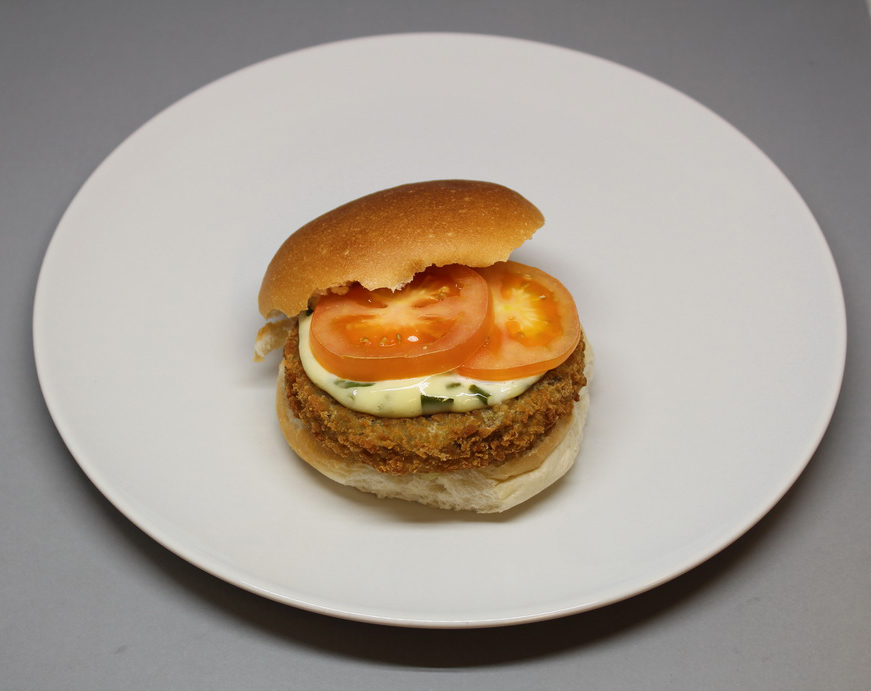

Supplement: Supplementary file 1 [file foods-11-00972-s001.zip › supplementary material/supplementary file S1/50.jpg]

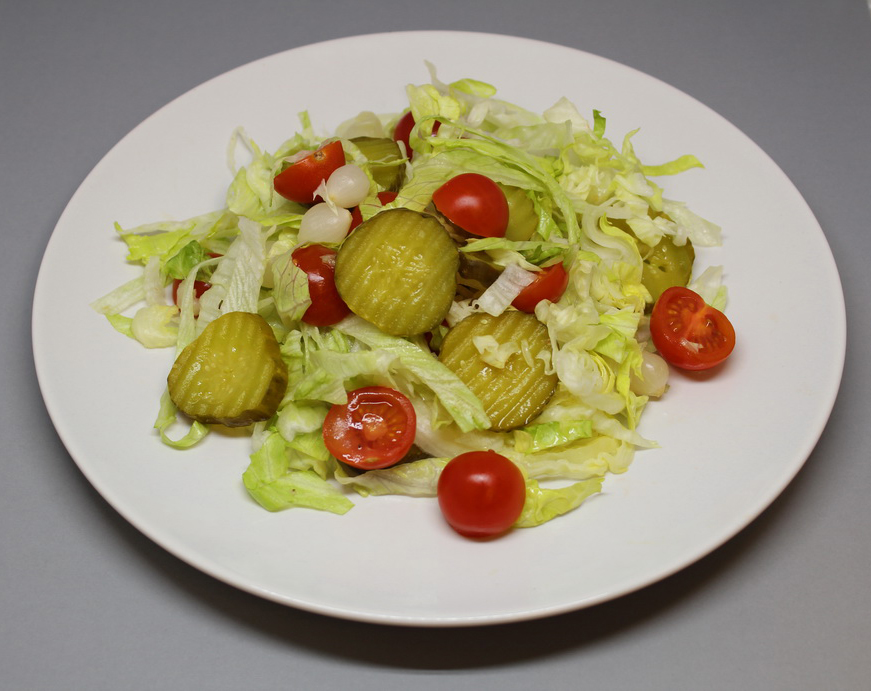

Supplement: Supplementary file 1 [file foods-11-00972-s001.zip › supplementary material/supplementary file S1/6.jpg]

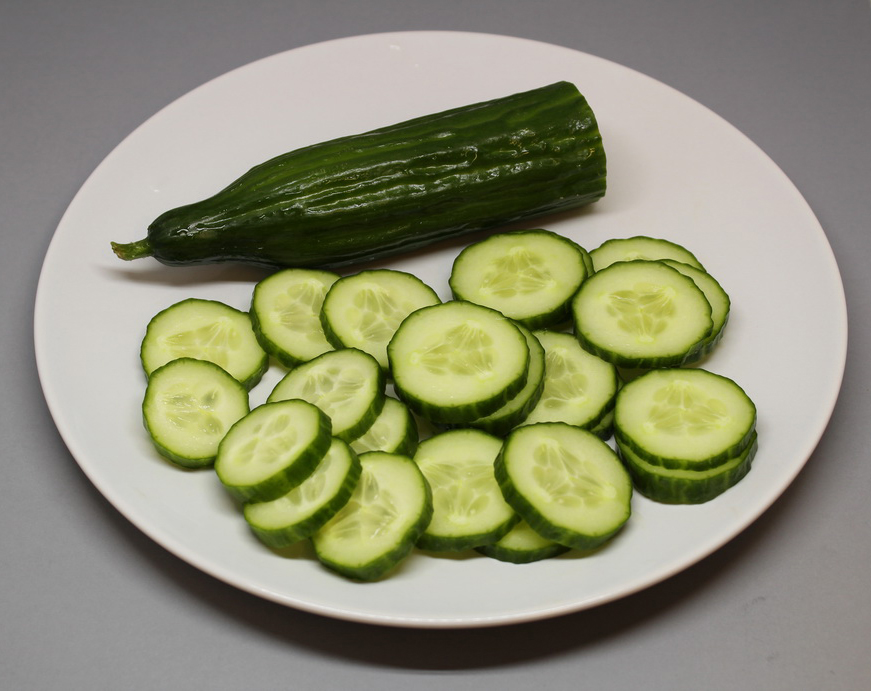

Supplement: Supplementary file 1 [file foods-11-00972-s001.zip › supplementary material/supplementary file S1/8.jpg]
